# Ketogenic Diet and Its Potential Role in Preventing Type 2 Diabetes Mellitus and Its Complications: A Narrative Review of Randomized Controlled Trials

**DOI:** 10.7759/cureus.66419

**Published:** 2024-08-08

**Authors:** Ali H Alluwyam, Edric D Estrella

**Affiliations:** 1 Department of Preventive Medicine, Saudi Board of Preventive Medicine, Al-Ahsa, SAU; 2 Department of Public Health, College of Applied Medical Sciences, King Faisal University, Al-Ahsa, SAU

**Keywords:** rct, complication, prevention, diabetes type 2, ketogenic diet

## Abstract

Diabetes mellitus is a global health crisis affecting millions. Nutrition plays a vital role in its management and prevention. While carbohydrate reduction is beneficial for glycemic control, various dietary approaches exist. The ketogenic diet, characterized by very low carbohydrate intake, has shown promise in weight management and blood sugar control. However, its potential for preventing type 2 diabetes mellitus (T2DM) remains largely unexplored.

To evaluate the ketogenic diet's potential in preventing T2DM, this review searched the PubMed database for studies published between 2013 and 2023. Findings suggest that the diet can effectively aid weight loss and improve blood glucose levels. Some evidence indicates reduced reliance on diabetes medications. However, effects on cholesterol levels are inconsistent, and long-term adherence challenges exist. Additionally, potential micronutrient deficiencies and safety concerns require careful consideration.

While the ketogenic diet offers potential benefits, further research is needed to establish its efficacy and safety as a long-term prevention strategy for T2DM. However, the results of the present study indicate the need for further research in this area, utilizing rigorous methodology.

## Introduction and background

Diabetes mellitus (DM) is a chronic, metabolic disease characterized by hyperglycemia due to a decrease in insulin production or insulin sensitivity that eventually leads to severe damage to many of the body's systems [1]. The International Diabetes Federation (IDF) Diabetes Atlas 10th edition 2021 estimates that 537 million adults (20-79 years) suffer from DM, representing approximately one in 10 individuals. Unfortunately, this figure is predicted to rise to 643 million by 2030 and 783 million by 2045. Diabetes will be responsible for 6.7 million deaths in 2021, or around one death every five seconds. Five hundred and forty-one million adults have impaired glucose tolerance (IGT), which predisposes them to a higher risk of type 2 diabetes mellitus (T2DM) [2].

Overall, for the optimal management and prevention of diabetes especially for the prevention of DM complications, nutrition therapy plays an integral role [3]. Evidence supports the idea that reducing overall carbohydrate intake to improve glycemia may be applied to various eating patterns that meet individual needs and preferences [4]. The ketogenic diet is one of the well-known eating patterns used to manage diabetes risk factors such as obesity and glycemic control. The keto diet is described by *JAMA Network* as "A diet that restricts carbohydrate intake to less than 25 to 50 grams per day in an attempt to enhance tissues to use fat or ketones (acids produced by the liver) as fuel during caloric restriction." Ketogenic diets typically recommend that only 5% of calories come from carbohydrates, along with 75% from fat and 20% from protein [5].

A systematic review by Caprio et al. (2019) recommends considering the ketogenic diet to succeed in early glycemic control, especially in obese patients in the early disease stage. In addition, they recommend using the ketogenic diet to reduce the use of glucose-lowering agents [6]. A 2020 meta-analysis by Yuan and colleagues investigated the impact of the ketogenic diet on blood sugar control, insulin resistance, and cholesterol levels in people with T2DM. By examining 13 relevant studies, they found that the keto diet can effectively lower fasting blood sugar, reduce haemoglobin A1c, improve cholesterol levels, and decrease body mass index (BMI) [7]. In another systematic review by Tinguely et al. (2021), they studied the effects on glucose control, the use of glucose-lowering medication, weight, lipids, and kidney and liver function, in addition to adherence and feasibility. They state that the keto diet seems like a promising interventional diet to improve glycemic control in patients with T2DM. Furthermore, a dietitian and dedicated physicians should supervise and support the ketogenic diet to prevent adverse effects and enhance adherence [8].

The previous reviews vary in design quality; some included mixed study designs, and in others, T2DM was not the main outcome. Eventually, after reviewing the literature, we noticed that gaps in research regarding the ketogenic diet's effect on T2DM were long-term safety, adherence, tolerability, and the need for high-quality randomized controlled trials (RCTs). While some studies have explored aspects of the ketogenic diet and diabetes, direct evidence of its preventive role in T2DM remains scarce. The objective of this narrative review is to synthesize the available evidence on the effects of the ketogenic diet on T2DM prevention or its complications.

## Review

Methods

**Figure 1:**
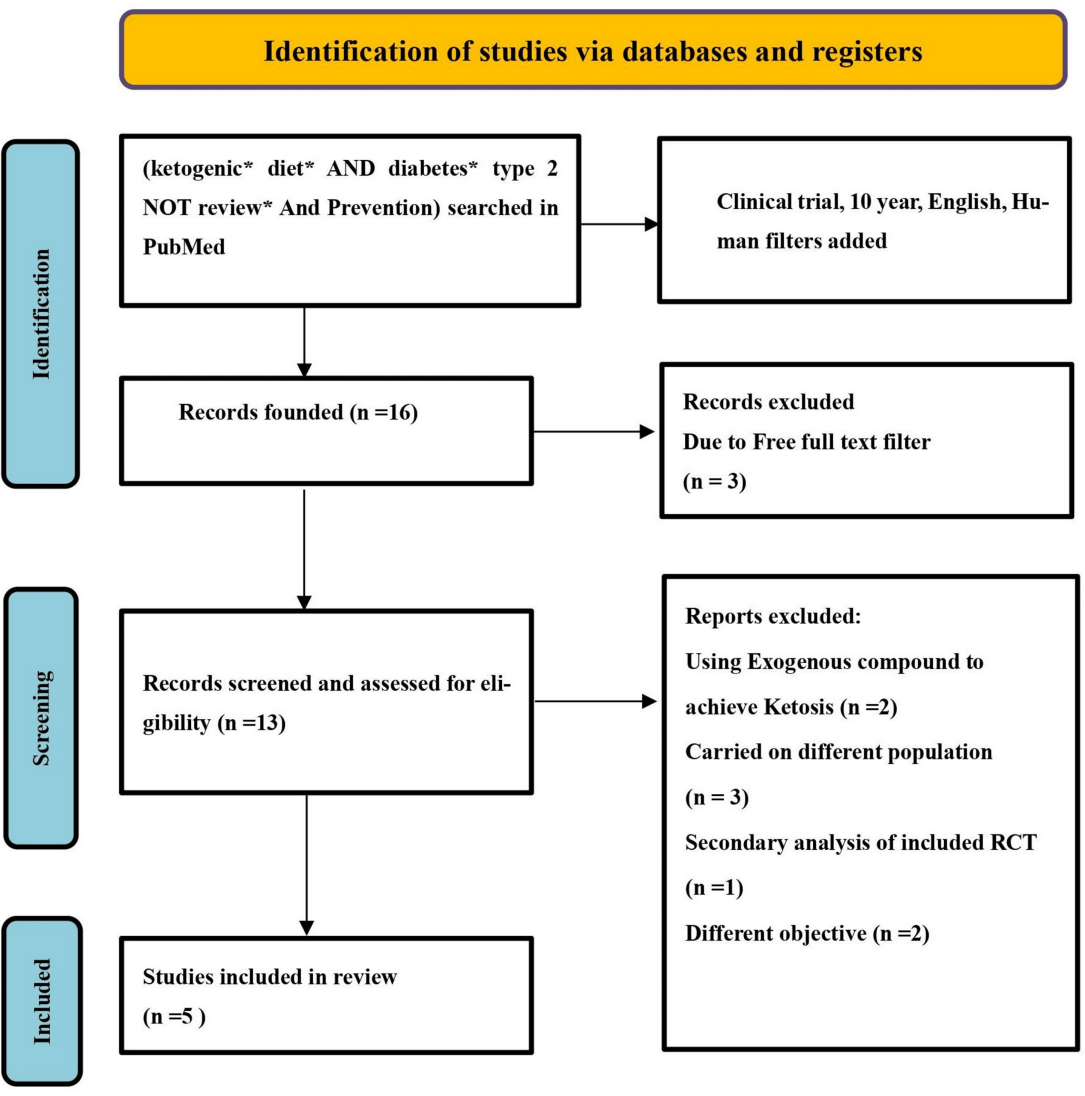
Flowchart of the Study Selection Process RCT: randomized controlled trial

PubMed was searched for studies published in the last 10 years (2013-2023) using the terms "ketogenic," "ketogenic diet," "Ketogenic* Diet*," "Diabetes*," "diabetes type 2," "Prevention". Searching PubMed with these keywords, ketogenic diet* AND diabetes* type 2 NOT review* with the following filters: Applying the human, last 10 years, and English filters yielded 136 studies, while the clinical trial filter yielded only 16. The free full-text filter excluded three studies, resulting in 13 studies. Five studies were excluded; two of them were due to the administration of an external compound to achieve ketosis exogenously, three of them carried over different populations (polycystic ovary syndrome patients, psychiatric patients, and cancer patients); one of them was a secondary analysis of an RCT that we included; and the last two's objective was to determine whether reducing hyperglycemia by following a low-carb, high-fat (LCHF) diet could lower inflammatory markers and improve sleep in T2DM people. The reviewer includes studies that are human studies, in English; RCTs on diabetic type 2 subjects, or prediabetics; and adult populations aged ≥18, studying the ketogenic diet as a management or preventive measure. The reviewer ends up with five relevant RCTs from PubMed. The study selection process is outlined in Figure 1.

Results

The results and characteristics of studies included in this review are summarized in Table 1.

**Table 1:** Summary of Characteristics of Included Studies * Diet: Olive oil, butter, eggs, pork, salmon, saury, sardines, broccoli, avocado. Daily: carbs 30-50g, protein 60g, fat 130g, total 1500±50 kcal [12]. ** No food limits. Daily: carbs 250-280g, protein 60g, fat 20g, total 1500±50 kcal [12]. *** Carbs: 20-50g/day, protein: 1.5g/kg ideal body weight/day. Remaining calories from fat [13]. **** Plant-based: vegetables, legumes, fruits, grains, nuts, seeds, fish, olive oil, dairy. No added sugars or refined grains [13]. RCT: randomized controlled trial

Author	Country	Year	Title	Population	Intervention	Comparison	Study Design	Sample Size	Duration	Conclusion
Goday et al. [9]	Spain	September 2016	Short-term safety, tolerability and efficacy of a very low-calorie-ketogenic diet interventional weight loss program versus hypocaloric diet in patients with type 2 diabetes mellitus	Individuals between the ages of 30 and 65 diagnosed with type 2 diabetes and a body mass index ranging from 30 to 35	Very low-carbohydrate ketogenic diet (VLCK diet)	Standard low-calorie diet	RCT, open-label, multi-centric	89	Four months	The study revealed that individuals with type 2 diabetes who followed a VLCK diet lost significantly more weight and experienced better blood glucose control compared to those on a standard calorie-restricted plan. The VLCKD program was also safe and well-tolerated by the participants.
Saslow et al. [10]	USA	February 2017	An online intervention comparing a very low-carbohydrate ketogenic diet and lifestyle recommendations versus a plate method diet in overweight individuals with type 2 diabetes: a randomized controlled trial	Type 2 diabetic ≥18 years with a body mass index of ≥25	VLCK diet and lifestyle recommendations	American Diabetes Association's “Create Your Plate” Diet	RCT, pilot feasibility study	25	32 weeks	Participants with type 2 diabetes achieved better glycemic control and greater weight loss when assigned to a VLCK diet with a lifestyle intervention delivered online, compared to those following a conventional low-fat diabetes diet program also delivered online.
Saslow et al. [11]	USA	December 2017	Twelve-month outcomes of a randomized trial of a moderate-carbohydrate versus very low-carbohydrate diet in overweight adults with type 2 diabetes mellitus or prediabetes	≥18 years, (BMI ≥ 25), glycated haemoglobin (HbA1c) level over 6.0%	VLCK diet	Moderate-carbohydrate, calorie-restricted, low-fat (MCCR) diet	RCT, parallel-group randomized (1:1) trial	34	One year	This year-long study found that a VLCK led to greater improvements in glycemic control (HbA1c reduction), weight loss, and decreased medication use compared to an MCCR diet in overweight or obese adults with pre-diabetes or type 2 diabetes.
Li et al. [12]	China	February 2022	The effect of periodic ketogenic diet on newly diagnosed overweight or obese patients with type 2 diabetes	Overweight or obese patients newly diagnosed as T2DM	Ketogenic diet*	Routine diet for diabetes**	RCT parallel group	60	Twelve weeks	This study suggests that intermittent keto dieting could benefit recently diagnosed diabetic patients who are overweight or obese. The diet could potentially manage weight, blood glucose, and cholesterol levels. However, long-term adherence is challenging.
Gardner et al. [13]	USA	May 2022	Effect of a ketogenic diet versus Mediterranean diet on glycated hemoglobin in individuals with prediabetes and type 2 diabetes mellitus: The interventional Keto-Med randomized crossover trial	≥18 years of age with a diagnosis of prediabetes or type 2 diabetes mellitus	A well-formulated ketogenic diet (WFKD)***	A Mediterranean-plus diet (Med-Plus) ****	RCT, Cross over	40	Twelve weeks on each diet	Both diets led to improvements in a key blood glucose measure (HbA1c) after 12 weeks, likely because they shared some features. One diet (WFKD) was more effective at lowering triglycerides, but it may also raise bad cholesterol (LDL) levels.

Effects on Glycemic Control

Glycemic control can be assessed using HbA1C levels, continuous glucose monitoring, or blood glucose monitoring. HbA1C is used in clinical trials to demonstrate the benefits of improved glycemic control [3]. In a pilot study in February 2017, they randomized overweight adults (BMI ≥ 25 kg/m^2^) who have T2DM and are not on diabetic medications other than metformin (HbA1c: 6.5%-9%) to 32-week online intervention (very low carbohydrate 20-50g per day ketogenic diet and lifestyle online program including mindful eating discussing the importance of physical activity and sleeping) (n=12) compared to American Diabetes Associations "Create Your Plate" Diet a low-fat diet that focuses on green vegetables, lean meat and somewhat limiting starchy and sweet foods(n=13). The study revealed that participants undergoing the intervention significantly lowered their HbA1c levels compared to the control group. The average HbA1c decrease was 0.8% in the intervention group (95% confidence interval (CI): -1.1% to -0.6%) versus 0.3% in the control group (95% CI: -0.6% to 0.0%). This difference was statistically significant (p=0.002) [10].

Saslow et al. (2017) conducted a year-long RCT involving 34 overweight or obese adults aged 18 or older with uncontrolled blood glucose levels (HbA1c > 6%). Participants were excluded if they used insulin or multiple medications for diabetes. The researchers randomly divided participants into two groups: one following a very low-carbohydrate ketogenic (VLCK) diet and the other adhering to a moderate-carbohydrate, calorie-restricted, low-fat (MCCR) diet. The study found that those on the ketogenic diet experienced a significantly greater drop in HbA1c levels compared to the other group. Average HbA1c levels decreased from 6.6% to 6.1% in the keto group, while they only decreased from 6.9% to 6.7% in the comparison group (p=0.007) [11].

A 2016 study compared a VLCK diet (carbohydrate <50g per day) to a standard low-calorie (LC) diet in 89 adults with T2DM and obesity. Participants were aged 30 to 65 and had a BMI between 30 and 35. After four months, the study found that the VLCK diet group experienced significantly greater reductions in HbA1c and improved blood glucose control compared to the standard diet group (p<0.05) [9].

In a 2022 RCT, Li et al. assigned 60 newly diagnosed adults (aged 16-50) with overweight or obese and had no history of hypoglycemic drug use to either a ketogenic diet (30-50g carbohydrates) or a standard diabetes diet. Both groups consumed approximately 1500 calories per day. Each group had 30 people for 12 weeks. Seven subjects withdrew from the study, six in the ketogenic diet group and one in the routine diet group due to adherence issues. Fasting blood glucose and HbA1c were reduced after intervention in both groups as compared to themselves (p<0.05); the reduction rate was significantly higher in the ketogenic diet group (p<0.05) [12].

The Keto-Med study, a 2022 interventional crossover trial involving adults with prediabetes or T2DM, compared a well-formulated ketogenic diet (WFKD) to a Mediterranean diet (Med-Plus). The Med-Plus diet is a modified Mediterranean diet that adheres to the Mediterranean diet but excludes added sugars and refined grains. Researchers found no significant difference in HbA1c reduction between the two diets WFKD: -9% (95% CI: -11% to -7%); Med-Plus: -7% (95% CI: -9% to -5%); (p=0.11) [13].

Effects on Weight and Anthropometric Measures

The pilot study conducted in February 2017 indicated that the intervention group members encountered significantly greater weight reduction compared to the control group. The average weight loss was 12.7 kg (95% CI: -16.1 to -9.2 kg) in the intervention group and 3.0 kg (95% CI: -7.3 to 1.3 kg) in the control group (p<0.001) [10]. A 2017 RCT by Saslow and colleagues found that participants on a VLCK lost significantly more weight compared to those on an MCCR diet. The average weight loss in the LCK group was 7.9 kg, compared to 1.7 kg in the MCCR group (p<0.001) [11]. A study done in 2016 found that both weight loss and waist circumference were greater in the VLCK diet group (both p<0.001). By the end of the study, more than 85% of the people who were on the VLCK diet had lost more than 10% of their starting weight [9]. RCTs conducted in February 2022 found similar results, which were for both ketogenic diet (KD) and routine diabetic diet groups. BMI and waist reduction were significantly higher in the ketogenic diet group (p=<0.05) [12]. A 2022 crossover study by Gardner and colleagues found that participants on the WFKD lost significantly more weight than those on the Med-Plus at the 12-week mark. The average weight loss was 6.9 kilograms (standard error of the mean, ±0.8 kg) for the WFKD group and 5.0 kilograms (standard error of the mean, ±0.8 kg) for the Med-Plus group (p=0.04) [13].

Effects on Diabetes-Related Medication

Saslow et al. (2017) discovered that people in the LCK group cut down on their diabetes medications more than those in the MCCR group. Six out of 10 people in the LCK group stopped taking the sulfonylureas or dipeptidyl peptidase-4 inhibitors; they were taking them at baseline, but only one out of six people in the MCCR group did so (p=0.005) [11].

Effects on Lipid Profile

In 2016, Goday et al. conducted an RCT and found that people on a VLCK diet and people on a low-calorie, low-fat diet did not have significantly different levels of total cholesterol (TC), low-density lipoprotein (LDL), or high-density lipoprotein (HDL). However, the VLCK group experienced a significantly lower triglyceride level compared to the low-calorie, low-fat group (p=0.004) [9]. An RCT done in China in February 2022, on the other hand, found that the lipid profile went down for both the ketogenic diet and routine diabetic diet groups (both were 1500 ± 50 calories), but the ketogenic diet group had significantly higher rates of TC, triglycerides (TG), LDL, and HDL reductions (p=0.05) [12]. In the Keto-Med crossover trial, WFKD led to a much greater drop in TG (p=0.02), but LDL levels rose significantly compared to a drop for Med-Plus (p=0.01) [13].

Adherence and Tolerability

Li et al. (2022) did a follow-up for willingness to adhere to the prescribed diet and found that the ketogenic diet group has a lower willingness compared to the diabetic diet, but 63% (19/24) of the ketogenic diet group are willing to stick to the ketogenic diet in the short term (p<0.05) [12]. Worth noting that six participants withdrew from the ketogenic diet due to adherence issues compared to one from the control group [12]. In the same way, the Keto-Med study found that both diet groups adhered to dietary plans equally after 12 weeks of follow-up, as shown on a scale from 1 to 10 (where 10 meant perfect adherent) [13].

Safety

A study from 2016 by Goday et al. found that kidney function markers (urine albumin-to-creatinine ratio, creatinine, and blood urea nitrogen) were not significantly different between people who were on a VLCK diet and those who were on an LC diet. While 91.1% of the VLCK group experienced ketosis, none of them presented with an RBG >250 mg/dL or pH <7.3 along with the ketosis. There were no significant differences in microalbuminuria between groups (p=0.156). However, uric acid and liver enzymes (alanine aminotransferase (ALT): 45.16 IU mL^−1^ compared to 26.85 IU mL^−1^, P<0.005; aspartate aminotransferase (AST): 38.53 IU mL^−1^ compared to 22.15 IU mL^−1^, P<0.001) were higher in the VLCK group at first but went back to normal by the end of the four-week study. Levels of sodium, potassium, chloride, calcium, and magnesium remained within normal ranges for both groups [9].

Li et al. (2022) reported higher rates of hypoglycemia in the ketogenic diet group during the initial four weeks compared to the control group. Specifically, there were 10 person-times of hypoglycemic symptoms and two person-times of severe hypoglycemia (blood glucose <3.9 mmol/L) in the keto group, versus 10 person-times of hypoglycemic symptoms and no hypoglycemia cases in the control group. However, both groups experienced no hypoglycemic events in the subsequent weeks. Additionally, uric acid levels increased in the ketogenic diet group but not in the control group, although this difference was not statistically significant [12].

In their 2022 Keto-Med crossover trial, Gardner et al. documented four adverse events. One participant experienced elevated ALT levels, possibly linked to the WFKD. Two other participants developed kidney infections and exacerbated eczema while on the WFKD, but these events were likely unrelated to the diet. Additionally, a transient ischemic attack occurred in a participant during the study follow-up while on the Mediterranean diet, which was deemed unrelated to the study [13].

Could the Ketogenic Diet Stand Against the Mediterranean Diet?

In the USA in 2022, a cross-over study compared the ketogenic and Mediterranean diets in adults with prediabetes or T2DM. Researchers randomly assigned 40 participants to follow each diet for 12 weeks. While both diets were low in carbohydrates and excluded added glucose and refined grains, the ketogenic diet was stricter, eliminating legumes, fruits, and whole grains. Both diets improved blood glucose control, as measured by HbA1c, but neither was significantly better than the other. The ketogenic diet not only effectively lowered TG but also increased LDL cholesterol and was associated with potential nutrient deficiencies due to its restrictive nature. Additionally, participants may have found the Mediterranean diet easier to sustain long-term. Overall, the study suggests that both dietary approaches can benefit blood glucose control, but the Mediterranean diet may offer a more balanced nutritional profile and better dietary adherence [13].

Could the Ketogenic Diet Prevent T2DM?

The risk of T2DM, heart disease, high blood pressure, and stroke increases if a person is overweight [14]. Lifestyle changes, including diet modification, exercise, and weight reduction, slow the progression of IGT to diabetes [15]. Lifestyle changes are, in general, beneficial and do not have serious adverse effects [15]. The ketogenic diet showed promising potential as it succeeded in reducing weight, improving glycemic control, and decreasing diabetic-related medication; however, this review is just opening the door for further studies with more rigorous research methodology.

Discussions

The findings of this review consistently demonstrate the efficacy of ketogenic diets in managing T2DM. A central theme across the included studies is the significant improvement in glycemic control, as evidenced by the reduction in HbA1c levels [9-13]. This effect is likely multifaceted and attributed to factors such as weight loss, increased insulin sensitivity, and potential alterations in gut microbiota composition. These results have been replicated in multiple reviews [16-18].

Weight management is another cornerstone of T2DM management, and the ketogenic diet has proven effective in inducing substantial weight loss [9-13]. This is crucial, as obesity is a primary risk factor for developing T2DM and its complications. The observed reductions in BMI and waist circumference across multiple studies underscore the diet's potential for combating weight-related metabolic disturbances. Several studies have confirmed these findings [16,17,19]. While the effects of ketogenic diets on lipid profiles present a more complex picture, it is evident that these diets can effectively lower triglyceride levels. However, the increase in LDL cholesterol in some studies warrants careful monitoring and consideration of individual risk factors [9,12,13]. It is essential to weigh the potential benefits of triglyceride reduction against the risks associated with elevated LDL cholesterol when making dietary recommendations. However, some reviews show improvement in lipid profile and cardiovascular risk [16,20].

The ability of ketogenic diets to reduce the need for diabetes medications is a promising finding [8,11]. This not only improves patient adherence but also has implications for healthcare costs. However, it is crucial to emphasize that medication reduction should be undertaken under strict medical supervision to prevent uncontrolled hyperglycemia.

Safety and tolerability are paramount considerations when evaluating dietary interventions. Although the ketogenic diet is generally well-tolerated, one cannot overlook the risk of adverse effects such as hypoglycemia, elevated liver enzymes, and potential nutrient deficiencies [9,12,13]. Under the guidance of healthcare professionals, implementing a diet can help mitigate these risks [21]. Long-term safety data is still limited, emphasizing the need for continued research in this area. Parry-Strong et al. (2022) support these findings [22]. It is interesting to consider the potential of ketogenic diets as a preventive measure for T2DM. Given the diet's efficacy in weight management and glycemic control, it may offer a valuable tool for individuals at risk of developing the disease. However, more research is needed to establish a definitive role for ketogenic diets in primary prevention.

It is essential to acknowledge the limitations of the current evidence base. The included studies vary in terms of participant characteristics, intervention duration, and outcome measures, making direct comparisons challenging. Additionally, the long-term effects of ketogenic diets on diabetes management and prevention remain to be fully elucidated.

Future research should focus on head-to-head comparisons of different ketogenic diet variations, the role of specific macronutrient ratios, and the impact of these diets on different diabetes subtypes. Long-term RCTs are needed to assess the sustained effects on glycemic control, weight management, and cardiovascular risk factors.

## Conclusions

Evidence supports the efficacy of ketogenic diets in improving glycemic control and promoting weight loss in individuals with T2DM. While these diets offer promising benefits, they should be implemented under medical supervision to address potential safety concerns and optimize outcomes. Further research is necessary to fully understand the long-term implications of ketogenic diets for diabetes management and prevention.
